# Supplementary material for: Young infants exhibit robust functional antibody responses and restrained IFN-γ production to SARS-CoV-2
Source: Cell Rep Med. 2021 Jun 9;2(7):100327. doi: 10.1016/j.xcrm.2021.100327 (PMC8188298; doi:10.1016/j.xcrm.2021.100327)
Supplement: Document S1. Figures S1–S3 [file mmc1.pdf]

Cell Reports Medicine, Volume *2*

Supplemental information

Young infants exhibit robust functional antibody responses and restrained IFN-γ production to SARS-CoV-2

Anu Goenka, Alice Halliday, Michaela Gregorova, Emily Milodowski, Amy Thomas, Maia Kavanagh Williamson, Holly Baum, Elizabeth Oliver, Anna E. Long, Lea Knezevic, Alistair J.K. Williams, Vito Lampasona, Lorenzo Piemonti, Kapil Gupta, Natalie Di Bartolo, Imre Berger, Ashley M. Toye, Barry Vipond, Peter Muir, Jolanta Bernatoniene, Mick Bailey, Kathleen M. Gillespie, Andrew D. Davidson, Linda Wooldridge, Laura Rivino, and Adam Finn

SUPPLEMENTAL INFORMATION

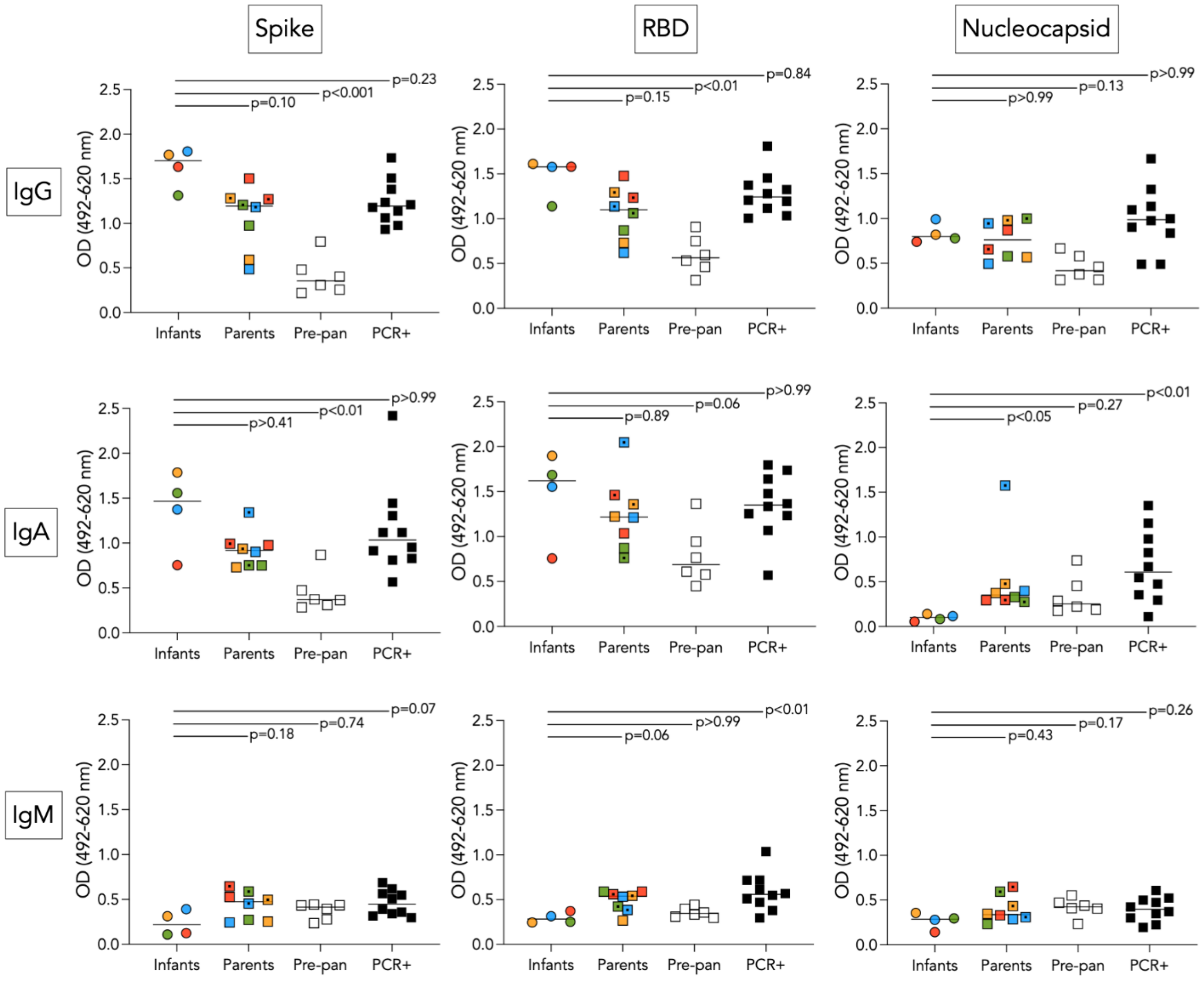

Figure S1. Robust antibody production to SARS-CoV-2 proteins across IgG and IgA isotypes in young infant sera. Related to Figure 1. Measurement of serum IgG, IgA and IgM against SARS-CoV-2 spike protein, receptor-binding domain (RBD) and nucleocapsid protein by ELISA, where background optical density (OD) at 620 nm was subtracted from signal OD at 492 nm.

All data points represent mean of technical duplicates. Individual families denoted by colour (1: red, 2: green, 3: blue; 4: orange); infants (coloured circles), fathers (coloured squares); mothers (coloured square with central marking); RT-PCR confirmed adult COVID-19 controls (black squares); pre-pandemic sera (clear squares). Significance determined by Kruskal-Wallis test with Bonferroni's correction for multiple comparisons.

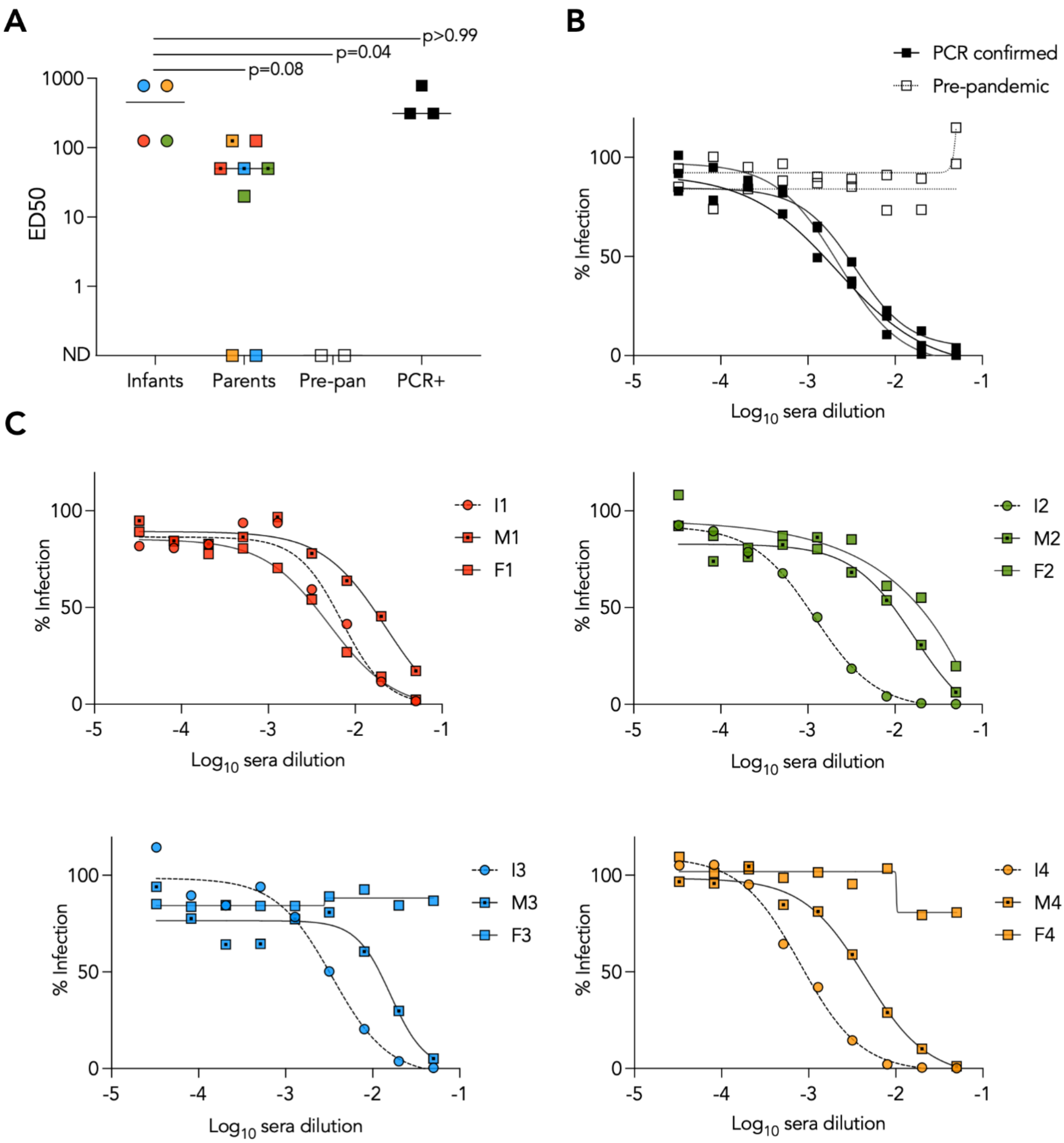

Figure S2. Robust neutralisation of SARS-CoV-2 by young infant sera. Related to Figure 1. (**A**) Serum neutralisation expressed as half maximal effective dose/dilution (ED50) of sera to achieve SARS-CoV-2 neutralisation. Significance determined by Kruskal-Wallis test with Bonferroni's correction for multiple comparisons; (**B**) Neutralisation of SARS-CoV-2 by serial dilutions of sera from pre-pandemic and RT-PCR confirmed adult controls; and (**C**) sera from each individual family. Neutralisation of SARS-CoV-2 measured by infection of Vero E6 cells with SARS-CoV-2 pre-incubated with decreasing concentrations of serum, where % infection is shown relative to virus-only control.

All data points represent mean of technical duplicates. Individual families denoted by colour (1: red, 2: green, 3: blue; 4: orange); infants (coloured circles), fathers (coloured squares); mothers (coloured square with central marking); RT-PCR confirmed adult controls (black squares); pre-pandemic controls (clear squares).

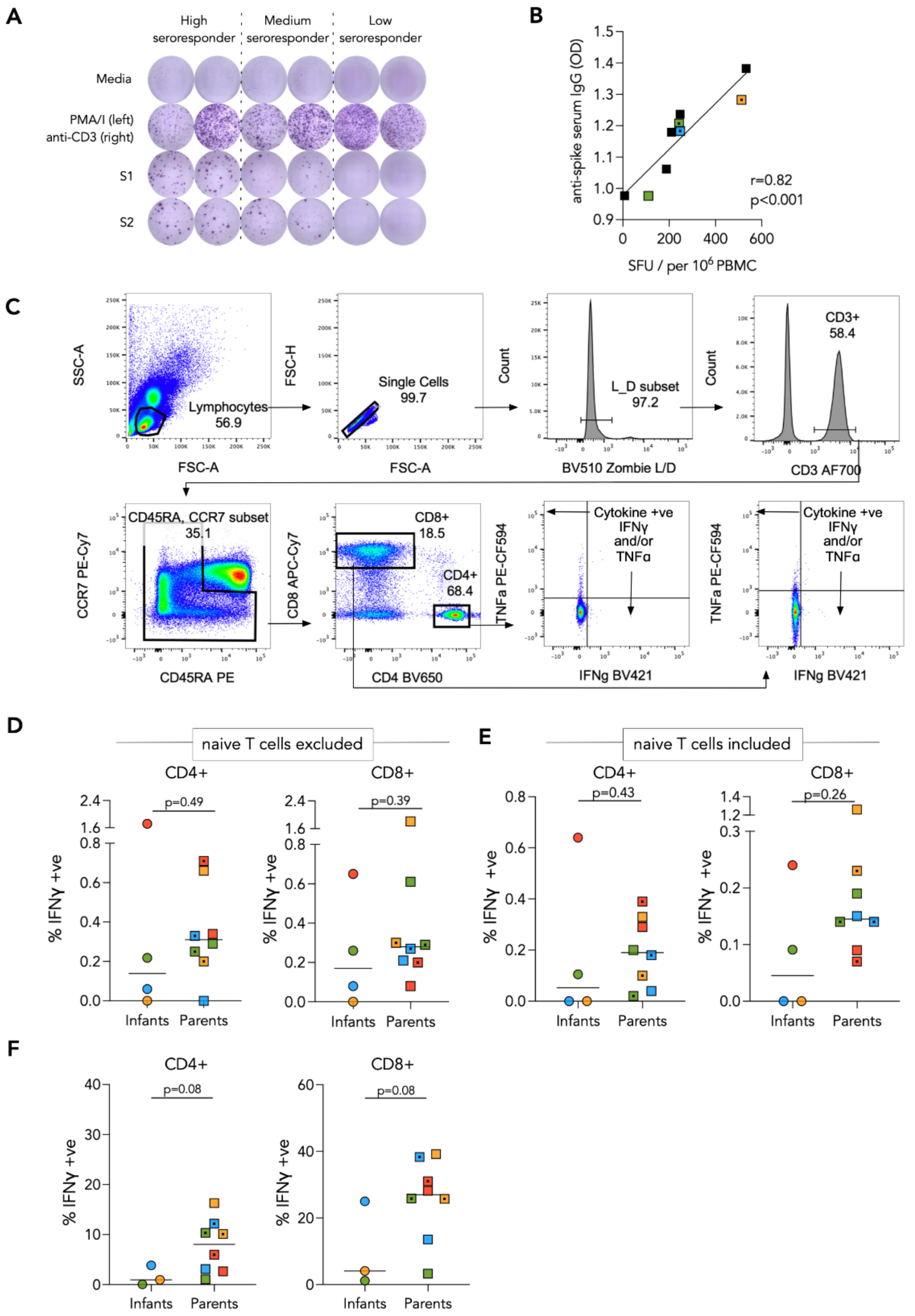

Figure S3. Detection of *ex vivo* cytokine production from PBMCs stimulated with SARS-CoV-2 peptide pools. Related to Figure 2. (**A**) Representative image of an ELISpot plate following 18h stimulation with SARS-CoV-2 peptide pools (S1, S2 at 2µg/mL), unstimulated (media) or positive control stimulus (PMA 1µg/mL/Ionomycin 10µg/mL or anti-CD3 antibody 0.1% v/v) of PBMCs from adults recovered from RT-PCR confirmed COVID-19; (**B**) Correlation of IFN-γ production measured by ELISpot and anti-spike IgG measured by ELISA in seropositive adults; (**C**) flow cytometry gating strategy for intracellular cytokine staining (ICS); (**D**) and (**E**) IFN-γ production of CD4+ and CD8+ T cells (with and without naïve $CD45RA^{+}$ $CCR7^{+}$ included respectively) measured by ICS of PBMCs following 5h *ex vivo* stimulation with SARS-CoV-2 peptide pools spanning spike (S1/S2) (1µg/mL). Significance determined by Mann Whitney U test**; (F)** IFN-γ production of CD4+ and CD8+ T-cells measured by ICS of PBMCs after 5h stimulation with PMA 1µg/mL/Ionomycin 10µg/mL. Significance determined by Mann Whitney U test.

Data points represent single observations (ICS) or mean of technical duplicates (ELISpot) with unstimulated signal subtracted in both ELISpot and ICS assays. Individual families denoted by colour (1: red, 2: green, 3: blue; 4: orange); infants (coloured circles), fathers (coloured squares); mothers (coloured square with central marking); and RT-PCR confirmed adult COVID-19 controls (black squares).
