# Supplementary material for: Young infants exhibit robust functional antibody responses and restrained IFN-γ production to SARS-CoV-2
Source: Cell Rep Med. 2021 Jun 9;2(7):100327. doi: 10.1016/j.xcrm.2021.100327 (PMC8188298; doi:10.1016/j.xcrm.2021.100327)
Supplement: Document S2. Article plus supplemental information [file mmc2.pdf]

Cell Reports
Medicine

Report

Young infants exhibit robust functional antibody responses and restrained IFN-γ production to SARS-CoV-2

Graphical abstract

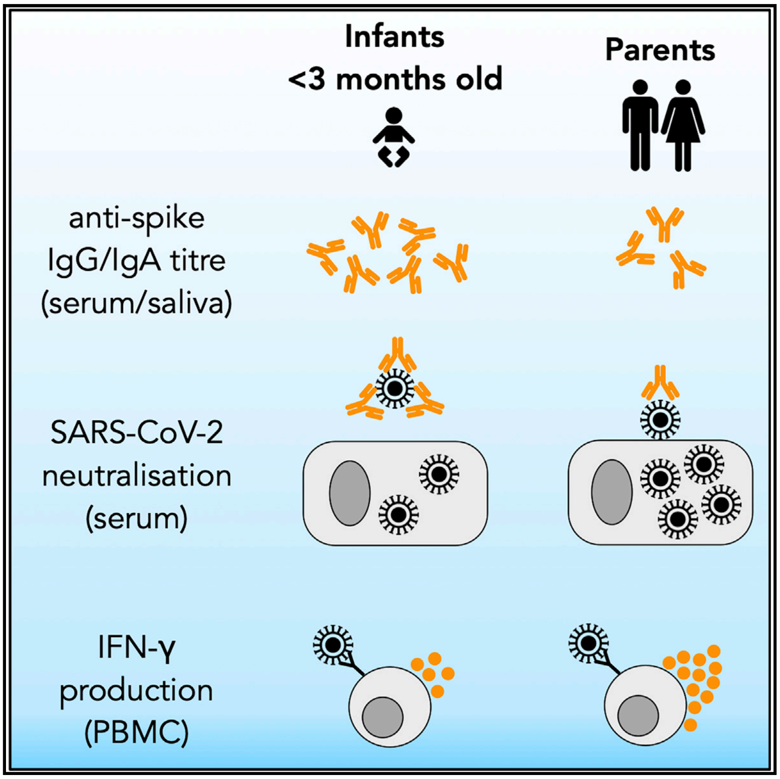

Highlights

- Distinct SARS-CoV-2 immune response of young infants compared with their parents
- High anti-spike IgG levels in sera and saliva of young infants
- Robust SARS-CoV-2 neutralization by sera of young infants
- Restrained spike-specific IFN-γ production by PBMCs of young infants

Authors

Anu Goenka, Alice Halliday, Michaela Gregorova, ..., Linda Wooldridge, Laura Rivino, Adam Finn

Correspondence

anu.goenka@bristol.ac.uk

In brief

Goenka et al. demonstrate that the SARS-CoV-2 immune response of young infants appears distinct compared with their parents. They show that infants exhibit relatively high serum (and saliva) levels of anti-spike IgG associated with robust SARS-CoV-2 neutralization but restrained cellular spike-specific IFN-γ production.

Goenka et al., 2021, Cell Reports Medicine 2, 100327
July 20, 2021 © 2021 The Author(s).
https://doi.org/10.1016/j.xcrm.2021.100327

CellPress

Cell Reports Medicine

CellPress
OPEN ACCESS

Report

Young infants exhibit robust functional antibody responses and restrained IFN-γ production to SARS-CoV-2

Anu Goenka,[1,2,13,14,15,*] Alice Halliday,[1,13] Michaela Gregorova,[1,13] Emily Milodowski,[3,13] Amy Thomas,[3] Maia Kavanagh Williamson,[1] Holly Baum,[4,5] Elizabeth Oliver,[1] Anna E. Long,[6] Lea Knezevic,[3] Alistair J.K. Williams,[6] Vito Lampasona,[7] Lorenzo Piemonti,[7] Kapil Gupta,[8] Natalie Di Bartolo,[5,8] Imre Berger,[5,8] Ashley M. Toye,[5,8,9,10] Barry Vipond,[11] Peter Muir,[11] Jolanta Bernatoniene,[2] Mick Bailey,[3] Kathleen M. Gillespie,[6] Andrew D. Davidson,[1] Linda Wooldridge,[3,13] Laura Rivino,[1,13] and Adam Finn[1,2,12,13]

[1]School of Cellular and Molecular Medicine, University of Bristol, Bristol, UK
[2]Department of Paediatric Immunology and Infectious Diseases, Bristol Royal Hospital for Children, Bristol, UK
[3]Bristol Veterinary School, University of Bristol, Bristol, UK
[4]School of Chemistry, University of Bristol, Bristol, UK
[5]Bristol Synthetic Biology Centre, University of Bristol, Bristol, UK
[6]Diabetes and Metabolism, Bristol Medical School, University of Bristol, Bristol, UK
[7]Diabetes Research Institute, IRCCS San Raffaele Scientific Institute, Milan, Italy
[8]School of Biochemistry, University of Bristol, Bristol, UK
[9]NIHR Blood and Transplant Research Unit in Red Blood Cell Products, University of Bristol, Bristol, UK
[10]Bristol Institute of Transfusion Science, NHS Blood and Transplant, Bristol, UK
[11]National Infection Service, Public Health England South West, Southmead Hospital, Bristol, UK
[12]School of Population Health Sciences, University of Bristol, Bristol, UK
[13]These authors contributed equally
[14]Twitter @anu_goenka
[15]Lead contact
*Correspondence: anu.goenka@bristol.ac.uk
https://doi.org/10.1016/j.xcrm.2021.100327

SUMMARY

Severe COVID-19 appears rare in children. This is unexpected, especially in young infants, who are vulnerable to severe disease caused by other respiratory viruses. We evaluate convalescent immune responses in 4 infants under 3 months old with confirmed COVID-19 who presented with mild febrile illness, alongside their parents, and adult controls recovered from confirmed COVID-19. Although not statistically significant, compared to seropositive adults, infants have high serum levels of IgG and IgA to SARS-CoV-2 spike protein, with a corresponding functional ability to block SARS-CoV-2 cellular entry. Infants also exhibit robust saliva anti-spike IgG and IgA responses. Spike-specific IFN-γ production by infant peripheral blood mononuclear cells appears restrained, but the frequency of spike-specific IFN-γ- and/or TNF-α-producing T cells is comparable between infants and adults. On principal-component analysis, infant immune responses appear distinct from their parents. Robust functional antibody responses alongside restrained IFN-γ production may help protect infants from severe COVID-19.

INTRODUCTION

The coronavirus disease 2019 (COVID-19) pandemic is responsible for unprecedented morbidity and mortality, particularly in the elderly, but significant disease appears rare in children.[1] Compared with older children, severe COVID-19 has been reported relatively more commonly in young infants.[1] Despite this, approximately one-fourth of young infants infected with severe acute respiratory syndrome-coronavirus-2 (SARS-CoV-2) are asymptomatic and there have been few reported deaths in this age group.[2] This is unexpected, given that early life is a period of rapid transition for the immune system that renders infants vulnerable to severe respiratory viral infections such as those caused by respiratory syncytial virus and influenza.[3,4] Few data are available describing SARS-CoV-2 immunity in infants younger than 3 months old. We therefore evaluated antibody and cellular immune responses in a small cohort of young infants recovered from COVID-19.

RESULTS AND DISCUSSION

Four infants younger than 12 weeks old presented with fever without an obvious clinical focus to Bristol Royal Hospital for Children (Bristol, UK) over a 4-week period in March 2020. Baseline characteristics of the infants (I1–I4), their mothers (M1–M4), and their fathers (F1–F4) are shown in Table 1. All of

© 2021 The Author(s).
This is an open access article under the CC BY license (http://creativecommons.org/licenses/by/4.0/).

Table 1. Characteristics of participants

	Infants, n = 4	Parents, n = 8	Adult COVID-19 controls, n = 10
**Age at presentation, median (range)**	7 wk (1–11 wk)	31 y (23–41 y)	31 y (24–39 y)
**Sampling interval post-symptom onset, median (range), days**	78 (35–91)	66 (34–91)	63 (30–100)
**Gender (male:female)**	2:2	4:4	5:5
**Ethnicity (white:Asian:Black)**	2:2:0	4:4:0	7:2:1
Clinical features, n (%)			
Asymptomatic	0 (0)	2 (25)	0 (0)
Fever	4 (100)	4 (50)	8 (80)
Cough	4 (100)	4 (50)	6 (60)
Coryza	4 (100)	1 (12.5)	3 (30)
Sore throat	N/A	1 (12.5)	2 (20)
Loss taste/smell	N/A	3 (37.5)	9 (90)
Gastrointestinal features	0 (0)	1 (12.5)	3 (30)
Symptoms >2 wk	0 (0)	2 (25)	4 (40)
Hospital admission, n (%)	4 (100)	0 (0)	0 (0)

the parents experienced COVID-19 symptoms in the days preceding the development of symptoms in their infants, except for 2 fathers (F3 and F4), who remained asymptomatic. The median age of the infants at presentation was 7 weeks (I1, 6 weeks; I2, 1 week; I3, 11 weeks corrected age; I4, 7 weeks). One infant was exclusively breastfed (I2), 1 was exclusively formula fed (I3), and 2 were mixed formula fed and breastfed (I1 and I4). There was no significant perinatal or medical history, except in 1 infant (I3) who was born at 28 weeks' gestation and did not suffer significant complications of prematurity but had been recently admitted to the hospital with rhinovirus bronchiolitis. Reduced peripheral lymphocyte counts of 1.2–2.1 × 10^9/L cells/mm^3 (normal range 3.3–10.3 × 10^9/L cells/mm^3) were observed in 2 infants (I1 and I2) but were normal in 1 infant (I4) and not measured in 1 infant (I3). C-reactive protein was measured in 3 infants (I1, I2, and I4) and was <1 mg/L (normal range <5 mg/L) in all these infants alkl. A transiently raised serum alanine aminotransferase with a peak of 207 U/L (normal range <33 U/L) was observed in 1 infant (I1). SARS-CoV-2 quantitative reverse transcription-polymerase chain reaction (qRT-PCR) was positive on nasopharyngeal swab in all 4 infants, with a median (range) cycle threshold value of 24.4 (22.0–29.9). Empirical treatment with intravenous antibiotics was commenced in 2 infants and discontinued at 36 h after negative blood and urine culture in 1 infant (I1), and after 14 days in the other (I2), from whom group B streptococcus was isolated from urine but not blood culture. None of the infants required oxygen therapy or feeding support and all of them exhibited symptom resolution within 2 days. Following recovery, peripheral blood and saliva were obtained for immunological analyses at a similar median interval after the onset of COVID-19 symptoms from infants (78 days), parents (66 days), and matched adult controls (63 days) who had recovered from qRT-PCR-proven COVID-19 (Table 1).

Human coronavirus infections typically result in the production of antibodies after 11–20 days that can persist for many months, some of which have neutralizing activity and correlate with protection against re-infection.[5] As such, serological assays have played a pivotal role in developing our understanding of adaptive and potentially protective immune responses to SARS-CoV-2 infection. Infants have been shown to produce broadly neutralizing antibodies rapidly to some viral infections, including HIV,[6] but typically generate lower systemic and mucosal antibody titers to other respiratory infections compared with adults.[7,8] In this study, we measured antibody responses to SARS-CoV-2 antigens using the luciferase immunoprecipitation system (LIPS) and an enzyme-linked immunosorbent assay (ELISA). All 4 infants exhibited robust serum immunoglobulin G (IgG) responses to the SARS-CoV-2 spike protein and its receptor-binding domain (RBD) (Figures 1A, 1B, and S1). Although infants' serum concentrations of anti-spike/RBD IgG appeared higher than those of their parents and qRT-PCR confirmed adult controls, the difference was not statistically significant after adjustment for multiple comparisons. Concentrations of serum IgG directed against SARS-CoV-2 nucleoprotein were low but above pre-pandemic levels for infants and their parents (Figures 1B and S1). Serum antibodies to SARS-CoV-2 antigens were not detected in the 2 asymptomatic parents. Relatively high serum IgA responses to spike and RBD were detected in 3 of the 4 infants (Figure S1). None of the infants and 6 of the parents/qRT-PCR-confirmed adult controls had SARS-CoV-2 specific IgM serum antibody titers clearly above those in pre-pandemic sera (Figure S1). Infants also exhibited robust salivary anti-spike IgG and IgA responses (Figures 1C and 1D). The infant IgA response may reflect endogenous antibody production rather than acquisition from maternal breast milk, because the exclusively formula-fed infant (I3) exhibited relatively high IgA titers compared with the more modest titers of the exclusively breastfed infant (I2) (Figures 1D and S1). A virus neutralization assay confirmed that the high anti-spike/RBD IgG titers in infant sera mirrored their functional ability to block SARS-CoV-2 entry into cells (Figures 1E, 1F, and S2). This is consistent with other reports demonstrating a direct relationship between high anti-spike/RBD titers and functional antibody responses in adults.[9] Thus, infants mounted robust and functional systemic and mucosal antibody responses to SARS-CoV-2 spike/RBD suggestive of clinically protective immunity.[10]

Alongside antibodies, T cells directed against SARS-CoV-2 have been observed in convalescent individuals.[11] Since interferon-γ (IFN-γ) has a key function in antiviral cell-mediated immunity,[11] we measured its production by peripheral blood mononuclear cells (PBMCs) stimulated with peptide pools spanning SARS-CoV-2 proteins using an enzyme-linked immunoabsorbent spot (ELISpot) assay (Figure S3A). Like others,[11] we observed a significant correlation ($r = 0.82$, $p < 0.001$) between the concentration of serum anti-spike IgG and IFN-γ production by PBMCs in response to stimulation by spike peptide pools among the seropositive adults recovered from COVID-19 (Figure S3B). Production of IFN-γ by PBMCs from infants and parents (alongside 4/5 PCR-proven adult COVID-19 controls) was

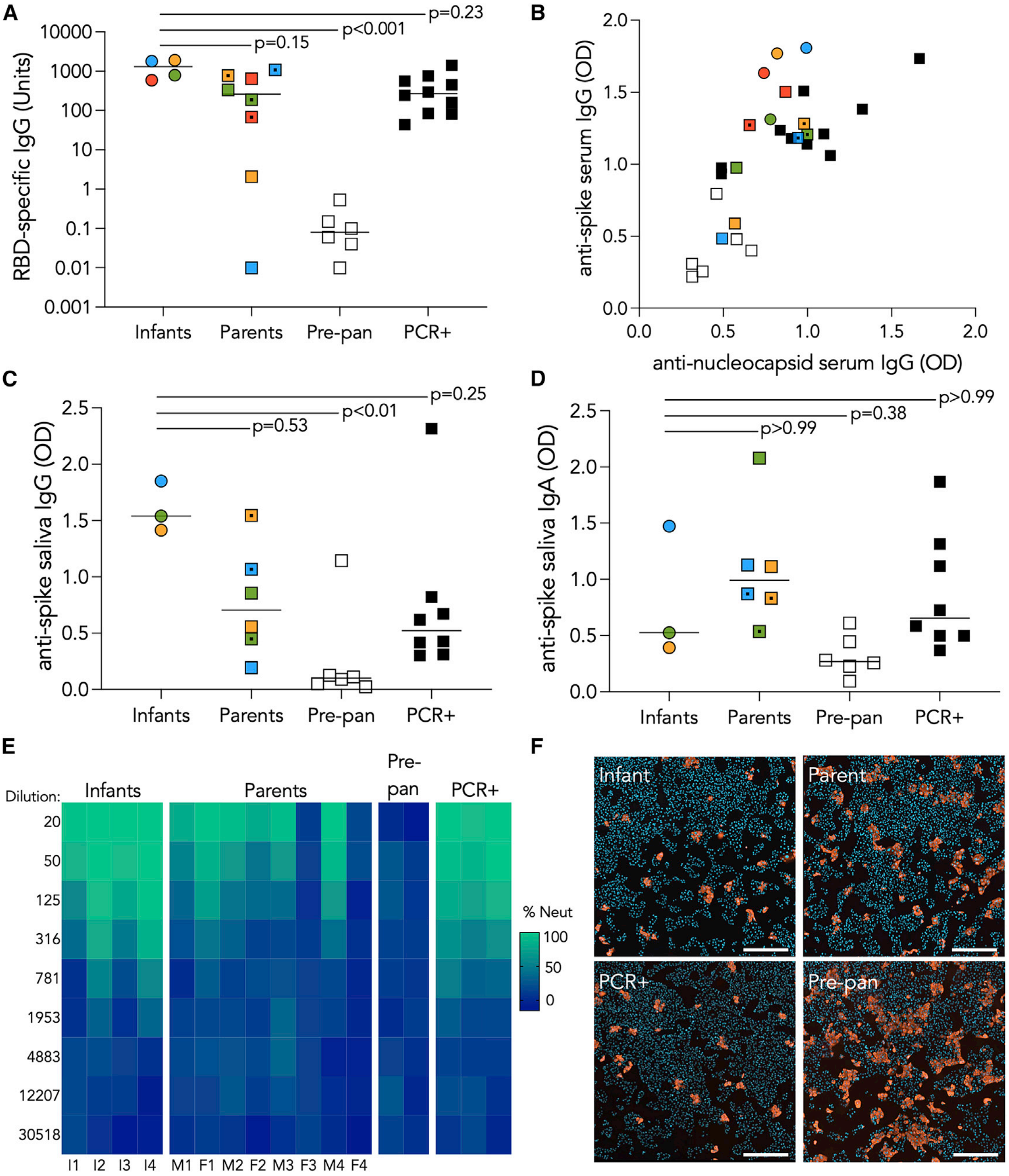

Figure 1. Robust and functional antibody response to SARS-CoV-2 in young infants

(A) Serum anti-RBD IgG measured using luciferase immunoprecipitation system (LIPS) assay expressed in units (derived from a pooled internal serum standard).

(B) Serum anti-spike IgG and anti-nucleocapsid IgG titers measured by ELISA; background optical density (OD) at 620 nm was subtracted from signal OD at 492 nm and corrected for average blank wells.

(C and D) Saliva anti-spike IgG (C) and (D) saliva anti-spike IgA (right) measured by ELISA; background optical density (OD) at 570 nm was subtracted from signal OD at 450 nm.

(E) Serum neutralization of SARS-CoV-2 measured by infection of Vero E6 cells with SARS-CoV-2 pre-incubated with decreasing concentrations of serum from infants (I1–I4), mothers (M1–M4), and fathers (F1–F4); adults recovered from RT-PCR confirmed COVID-19; and pre-pandemic sera, in which color intensity represents neutralization (i.e., percentage of infected cells relative to control wells containing virus only [no sera]).

(F) Representative immunofluorescence images of assay described in (D) with 1:125 dilution of sera from an infant (I2), parent (M2), pooled sera from RT-PCR-confirmed COVID-19 controls, and pre-pandemic samples, in which the nucleic acid of Vero E6 cells is stained by DAPI (blue) and SARS-CoV-2 is visualized with anti-nucleocapsid antibody (Rockland, 200-401-A50) and an Alexa Fluor 568 conjugated secondary antibody. Images were acquired and analyzed using the ImageXpress Pico system. Scale bar represents 500 μm.

(legend continued on next page)

detected following stimulation with spike peptide pools (Figure 2A). The 2 asymptomatic parents exhibited IFN-γ production (Figure 2A), which has been described in seronegative individuals and may represent SARS-CoV-2 exposure or cross-reactive T cell immunity from seasonal coronaviruses.[12,13] To further explore the antigen-specific cytokine production and its cellular source in infants, we measured IFN-γ and tumor necrosis factor α (TNF-α) production by $CD4^+$ and $CD8^+$ T cells using flow cytometric intracellular cytokine staining (ICS) following *ex vivo* stimulation of PBMCs with peptide pools spanning SARS-CoV-2 proteins (Figure S3C). Comparable frequencies of cytokine positive $CD4^+$ and $CD8^+$ T cells (defined as IFN-γ and/or TNF-α^+) were detectable among infants' and parents' PBMCs following stimulation with spike and membrane/nucleocapsid peptide pools (Figures 2B and 2C). Given the low magnitude of infant cellular responses we observed *ex vivo*, compared with relatively high infant anti-SARS-CoV-2 antibody titers, we sought to determine their T cell antigen specificity by *in vitro* expansion with SARS-CoV-2 peptide pools.[14] Of the 3 infants from whom we had a sufficient yield of PBMCs, all of them exhibited a significant expansion of $CD4^+$ T cells reactive to peptide pools spanning spike as well as M/N protein pools, suggestive of antigen specificity (Figures 2D–2F). Interestingly, infants' PBMCs appeared to exhibit a lower production of IFN-γ in response to spike protein compared with adults' by both ELISpot (Figure 2A) and ICS (Figures S3D and S3E), although the difference was not statistically significant after adjustment for multiple comparisons. These apparent differences may be representative of the well-documented and generalized decreased type 1 cytokine-producing ability of infant T cells,[15] which we also observed in response to mitogen stimulation (Figure S3F). Assessed by principal-component analysis, the antibody and cellular immune response to SARS-CoV-2 in young infants collectively appeared distinct from those of their parents, despite the lack of statistical significance in individual assays after adjustment for multiple comparisons (Figure 2G).

These data suggest that the mild clinical course of COVID-19 reported in young infants may be associated with robust functional antibody responses and restrained IFN-γ production. Describing the molecular mechanisms underlying the mild course of COVID-19 in infants during their period of vulnerability to other severe respiratory viral infections and contrasting them with those seen in severely affected adults may help explain the pathogenesis of severe COVID-19.

LIMITATIONS OF THE STUDY

There are several limitations of our study. This is a small cohort and participants underwent sampling at a single time point only. As well as confirming our observations in a larger cohort of participants, it would be valuable in future studies to study both innate and adaptive responses in infants compared with adults, in the acute phase of COVID-19 and by longitudinal observations in convalescence. The present study is also restricted to individuals recovering from mildly symptomatic COVID-19, therefore potentially not representative of the significant proportion of young infants and adults with asymptomatic infection. In addition, we were unable to assess the neutralizing capacity of the mucosal antibody due to the low sample volume and had insufficient PBMCs to definitively demonstrate their antigen specificity by tetramer staining. Comparing infant and adult T cell responses to a broader range of epitopes such as non-structural SARS-CoV-2 peptides would be of interest in future studies.[11,13,14]

STAR★METHODS

Detailed methods are provided in the online version of this paper and include the following:

- KEY RESOURCES TABLE
- RESOURCE AVAILABILITY
  - Lead contact
  - Materials availability
  - Data and code availability
- EXPERIMENTAL MODEL AND SUBJECT DETAILS
  - Human subjects and samples
  - Cell lines
- METHOD DETAILS
  - Protein production for ELISA
  - Serum ELISA
  - Saliva ELISA
  - Luciferase immunoprecipitation system (LIPS)
  - Virus neutralisation assay
  - Synthetic peptides
  - ELISpot
  - *Ex vivo* stimulation, intracellular cytokine staining and flow cytometry
  - *In vitro* expansion of T cells with SARS-CoV-2 peptides
- QUANTIFICATION AND STATISTICAL ANALYSIS

SUPPLEMENTAL INFORMATION

Supplemental information can be found online at https://doi.org/10.1016/j.xcrm.2021.100327.

ACKNOWLEDGMENTS

We dedicate this manuscript to the memory of our colleague and co-author Alistair J.K. Williams, whose enthusiasm and meticulous attention to detail played a key role in the development of the suite of COVID-19 serological assays developed at the University of Bristol. We are very grateful to the families and control individuals who participated in the study. We thank the Bristol UNCOVER team for helpful discussions during the execution of this work and the preparation of this manuscript. We are also grateful to the Diabetes and Metabolism Team for testing serum samples using the LIPS method. We also thank Jill King, Jane Metz, Charlie Plumptre, Rachael Barr, Begonia Morales-Aza, Lucy Collingwood, and Jenny Oliver for their work in assisting

Data points represent means of technical duplicates for serum assays or single observations for saliva ELISA. Individual families denoted by color (1: red, 2: green, 3: blue, 4: orange); infants (colored circles), fathers (colored squares); mothers (colored square with central marking); RT-PCR-confirmed adult COVID-19 controls (black squares); and pre-pandemic sera (clear squares). Significance determined by Kruskal-Wallis test with Bonferroni's correction for multiple comparisons.

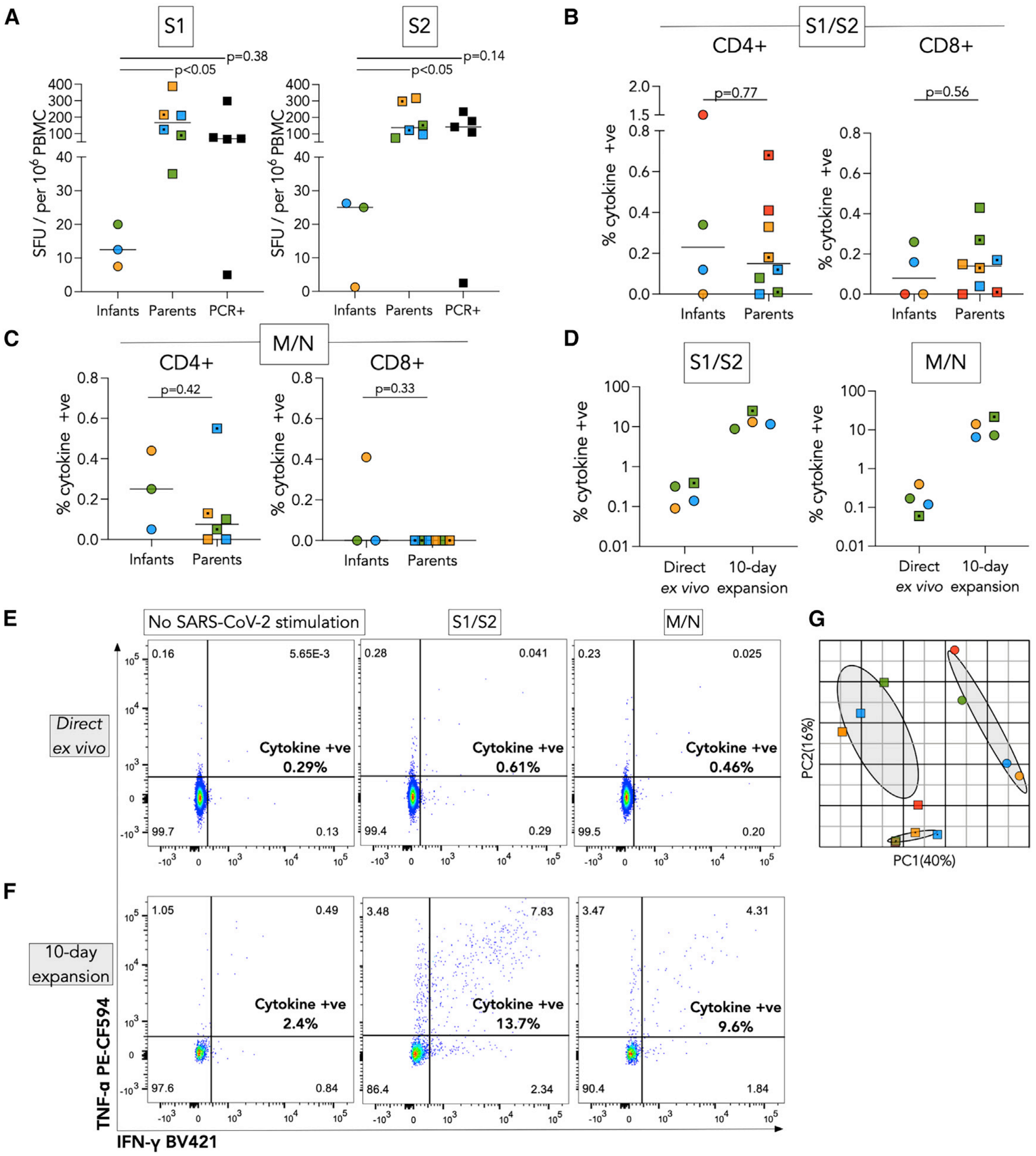

Figure 2. Young infants exhibit distinct cellular and antibody immune responses to SARS-CoV-2

(A) IFN-γ production measured by ELISpot following 18-h *ex vivo* stimulation of PBMCs with SARS-CoV-2 spike (S1 and S2) peptide pools (2 μg/mL). Significance determined by Kruskal-Wallis test with Bonferroni's correction for multiple comparisons.

(B and C) Proportion of cytokine (TNF-α and/or IFN-γ)-producing $CD4^+$ and $CD8^+$ T cells (naive $CD45RA^+$ $CCR7^+$ excluded) measured by intracellular cytokine staining (ICS) of PBMCs following 5-h *ex vivo* stimulation with SARS-CoV-2 peptide pools spanning spike (S1/S2) or membrane/nucleocapsid protein (1 μg/mL). Significance determined by Mann-Whitney *U* test.

(D) Proportion of cytokine-producing $CD4^+$ T cells measured by ICS following PBMC stimulation as described above (indicated as "Direct *ex vivo*") compared with *in vitro* expansion of PBMCs pulsed with peptide pools (5 μg/mL) for 45 min, followed by 10-day culture in the presence of interleukin-2 (IL-2) (20 IU/mL) and then re-stimulated for 5 h with SARS-CoV-2 peptide pools (indicated as "10-day expansion").

(E and F) Representative ICS plots of "Direct *ex vivo*" (E) and "10-day expansion" (F) samples from I2.

(G) Principal-component analysis incorporating data from assays measuring serum anti-spike/RBD/nucleocapsid IgG/IgM/IgA and viral neutralization; saliva IgG/IgA; IFN-γ production by total PBMCs (ELISpot); cytokine (TNF-α and/or IFN-γ)-positive $CD4^+$ and $CD8^+$ T cells (ICS) in infants and their parents; 95% confidence ellipses shown.

Data points represent single observations (ICS) or means of technical duplicates (ELISpot) with unstimulated signal subtracted in both ELISpot and ICS assays. Individual families denoted by color (1: red, 2: green, 3: blue, 4: orange); infants (colored circles), fathers (colored squares), mothers (colored square with central marking); RT-PCR confirmed adult controls (black squares), pre-pandemic controls (clear squares).

with participant recruitment and consent as part of Bristol Biobank. We thank Prof. Tao Dong (Oxford University) for kindly providing the spike peptide pools for the T-cell assays. We wish to acknowledge the assistance of Dr. Andrew Herman, Helen Rice, the University of Bristol Faculty of Biomedical Sciences Flow Cytometry Facility, and Debbie Shattock for technical assistance in performing the saliva antibody assays. This work was supported by funding from the Elizabeth Blackwell Institute (EBI) for Health Research, the University of Bristol, with funding from the University's alumni and friends (to A.G., L.W., L.R., A.H., and A.T.), the Grand Appeal charity (to A.G. and A.F.), Diabetes UK/JDRF (20/0006217, to K.M.G.), BBSRC/EPSRC Synthetic Biology Research Centre funding (BB/L01386X/1, to N.D.B. and A.M.T.), NHS Blood and Transplant (WP15-05, to N.D.B. and A.M.T.), the NIHR Blood and Transplant Research Unit in Red Cell Products (IS-BTU-1214-10032, to N.D.B. and A.M.T.), the UK Research and Innovation/Medical Research Council (MR/V027506/1, to A.D.D.), the Wellcome Trust GW4-CAT Fellowship (to E.M.), the Wellcome Trust (106115/Z/14/Z, to I.B.), and the Wellcome Trust Institutional Strategic Support Fund (204813/Z/16/Z, to A.F and A.H.). This manuscript presents independent research funded in part by the National Institute for Health Research (NIHR). The views expressed are those of the authors and not necessarily those of the NHS, the NIHR, or the Department of Health and Social Care. The support from the EBI and the University's alumni and friends also funded the purchase of the CTL ELISpot reader, ELISA microplate washer, and ImageXpress Pico Imaging platform, which were used in this study.

AUTHOR CONTRIBUTIONS

A.G. conceived the study and wrote the first draft of the manuscript with A.H. A.G. and J.B. collated the clinical data and led the collection of the samples. B.V. and P.M. performed SARS-CoV-2 qRT-PCR. E.O., L.K., and M.G. processed the blood samples. N.D.B., K.G., I.B., A.M.T., V.L., and L.P. produced the purified SARS-CoV-2 antigens for serology assays. E.O., A.H., A.T., and H.B. optimized and performed the ELISA assays. A.E.L., A.J.K.W., and K.M.G. optimized and performed the LIPS assays. M.K.W. and A.D.D. performed the neutralization assays. E.M. and L.W. performed the ELISpot assays. M.G. and L.R. performed the flow cytometry assays. A.T. and M.B. performed the statistical analysis and the principal-component analysis on the data. A.F., L.W., and L.R. supervised the work. All of the authors read, provided input on, and gave approval to the final manuscript.

DECLARATION OF INTERESTS

The authors declare no competing interests.

Received: January 25, 2021
Revised: April 13, 2021
Accepted: June 4, 2021
Published: June 9, 2021

REFERENCES

1. O'Driscoll, M., Ribeiro Dos Santos, G., Wang, L., Cummings, D.A.T., Azman, A.S., Paireau, J., Fontanet, A., Cauchemez, S., and Salje, H. (2021). Age-specific mortality and immunity patterns of SARS-CoV-2. Nature *590*, 140–145.
2. Trevisanuto, D., Cavallin, F., Cavicchiolo, M.E., Borellini, M., Calgaro, S., and Baraldi, E. (2021). Coronavirus infection in neonates: a systematic review. Arch. Dis. Child. Fetal Neonatal Ed. *106*, 330–335.
3. Jansen, A.G., Sanders, E.A., Hoes, A.W., van Loon, A.M., and Hak, E. (2007). Influenza- and respiratory syncytial virus-associated mortality and hospitalisations. Eur. Respir. J. *30*, 1158–1166.
4. Lambert, L., Sagfors, A.M., Openshaw, P.J., and Culley, F.J. (2014). Immunity to RSV in Early-Life. Front. Immunol. *5*, 466.
5. Huang, A.T., Garcia-Carreras, B., Hitchings, M.D.T., Yang, B., Katzelnick, L.C., Rattigan, S.M., Borgert, B.A., Moreno, C.A., Solomon, B.D., Trimmer-Smith, L., et al. (2020). A systematic review of antibody mediated immunity to coronaviruses: kinetics, correlates of protection, and association with severity. Nat. Commun. *11*, 4704.
6. Goo, L., Chohan, V., Nduati, R., and Overbaugh, J. (2014). Early development of broadly neutralizing antibodies in HIV-1-infected infants. Nat. Med. *20*, 655–658.
7. Brandenburg, A.H., Groen, J., van Steensel-Moll, H.A., Claas, E.C., Rothbarth, P.H., Neijens, H.J., and Osterhaus, A.D. (1997). Respiratory syncytial virus specific serum antibodies in infants under six months of age: limited serological response upon infection. J. Med. Virol. *52*, 97–104.
8. Holbrook, B.C., Hayward, S.L., Blevins, L.K., Kock, N., Aycock, T., Parks, G.D., and Alexander-Miller, M.A. (2015). Nonhuman primate infants have an impaired respiratory but not systemic IgG antibody response following influenza virus infection. Virology *476*, 124–133.
9. Robbiani, D.F., Gaebler, C., Muecksch, F., Lorenzi, J.C.C., Wang, Z., Cho, A., Agudelo, M., Barnes, C.O., Gazumyan, A., Finkin, S., et al. (2020). Convergent antibody responses to SARS-CoV-2 in convalescent individuals. Nature *584*, 437–442.
10. Addetia, A., Crawford, K.H.D., Dingens, A., Zhu, H., Roychoudhury, P., Huang, M.-L., Jerome, K.R., Bloom, J.D., and Greninger, A.L. (2020). Neutralizing Antibodies Correlate with Protection from SARS-CoV-2 in Humans during a Fishery Vessel Outbreak with a High Attack Rate. J. Clin. Microbiol. *58*, 1–11.
11. Peng, Y., Mentzer, A.J., Liu, G., Yao, X., Yin, Z., Dong, D., Dejnirattisai, W., Rostron, T., Supasa, P., Liu, C., et al.; Oxford Immunology Network Covid-19 Response T cell Consortium; ISARIC4C Investigators (2020). Broad and strong memory $CD4^+$ and $CD8^+$ T cells induced by SARS-CoV-2 in UK convalescent individuals following COVID-19. Nat. Immunol. *21*, 1336–1345.
12. Grifoni, A., Weiskopf, D., Ramirez, S.I., Mateus, J., Dan, J.M., Moderbacher, C.R., Rawlings, S.A., Sutherland, A., Premkumar, L., Jadi, R.S., et al. (2020). Targets of T Cell Responses to SARS-CoV-2 Coronavirus in Humans with COVID-19 Disease and Unexposed Individuals. Cell *181*, 1489–1501.e15.
13. Sekine, T., Perez-Potti, A., Rivera-Ballesteros, O., Strålin, K., Gorin, J.B., Olsson, A., Llewellyn-Lacey, S., Kamal, H., Bogdanovic, G., Muschiol, S., et al.; Karolinska COVID-19 Study Group (2020). Robust T Cell Immunity in Convalescent Individuals with Asymptomatic or Mild COVID-19. Cell *183*, 158–168.e14.
14. Le Bert, N., Tan, A.T., Kunasegaran, K., Tham, C.Y.L., Hafezi, M., Chia, A., Chng, M.H.Y., Lin, M., Tan, N., Linster, M., et al. (2020). SARS-CoV-2-specific T cell immunity in cases of COVID-19 and SARS, and uninfected controls. Nature *584*, 457–462.
15. Vigano, A., Esposito, S., Arienti, D., Zagliani, A., Massironi, E., Principi, N., and Clerici, M. (1999). Differential development of type 1 and type 2 cytokines and beta-chemokines in the ontogeny of healthy newborns. Biol. Neonate *75*, 1–8.
16. Clarke, E.T., Williams, N.A., Dull, P.M., Findlow, J., Borrow, R., Finn, A., and Heyderman, R.S. (2013). Polysaccharide-protein conjugate vaccination induces antibody production but not sustained B-cell memory in the human nasopharyngeal mucosa. Mucosal Immunol. *6*, 288–296.
17. Toelzer, C., Gupta, K., Yadav, S.K.N., Borucu, U., Davidson, A.D., Kavanagh Williamson, M., Shoemark, D.K., Garzoni, F., Staufer, O., Milligan, R., et al. (2020). Free fatty acid binding pocket in the locked structure of SARS-CoV-2 spike protein. Science *370*, 725–730.
18. Amanat, F., Stadlbauer, D., Strohmeier, S., Nguyen, T.H.O., Chromikova, V., McMahon, M., Jiang, K., Arunkumar, G.A., Jurczyszak, D., Polanco, J., et al. (2020). A serological assay to detect SARS-CoV-2 seroconversion in humans. Nat. Med. *26*, 1033–1036.
19. Berger, I., Fitzgerald, D.J., and Richmond, T.J. (2004). Baculovirus expression system for heterologous multiprotein complexes. Nat. Biotechnol. *22*, 1583–1587.
20. Secchi, M., Bazzigaluppi, E., Brigatti, C., Marzinotto, I., Tresoldi, C., Rovere-Querini, P., Poli, A., Castagna, A., Scarlatti, G., Zangrillo, A., et al.

(2020). COVID-19 survival associates with the immunoglobulin response to the SARS-CoV-2 spike receptor binding domain. J. Clin. Invest. *130*, 6366–6378.

21. Williams, A.J.K., Norcross, A.J., Chandler, K.A., and Bingley, P.J. (2006). Non-specific binding to protein A Sepharose and protein G Sepharose in insulin autoantibody assays may be reduced by pre-treatment with glycine or ethanolamine. J. Immunol. Methods *314*, 170–173.

22. Davidson, A.D., Williamson, M.K., Lewis, S., Shoemark, D., Carroll, M.W., Heesom, K.J., Zambon, M., Ellis, J., Lewis, P.A., Hiscox, J.A., and Matthews, D.A. (2020). Characterisation of the transcriptome and proteome of SARS-CoV-2 reveals a cell passage induced in-frame deletion of the furin-like cleavage site from the spike glycoprotein. Genome Med. *12*, 68.

23. Rivino, L., Kumaran, E.A., Thein, T.L., Too, C.T., Gan, V.C., Hanson, B.J., Wilder-Smith, A., Bertoletti, A., Gascoigne, N.R., Lye, D.C., et al. (2015). Virus-specific T lymphocytes home to the skin during natural dengue infection. Sci. Transl. Med. *7*, 278ra35.

24. Rivino, L., Kumaran, E.A., Jovanovic, V., Nadua, K., Teo, E.W., Pang, S.W., Teo, G.H., Gan, V.C., Lye, D.C., Leo, Y.S., et al. (2013). Differential targeting of viral components by CD4+ versus CD8+ T lymphocytes in dengue virus infection. J. Virol. *87*, 2693–2706.

STAR★METHODS

KEY RESOURCES TABLE

REAGENT or RESOURCE	SOURCE	IDENTIFIER
Antibodies		
Mouse anti-polyHistidine HRP conjugated antibody	Sigma-Aldrich	Cat#A7058; RRID: AB_258326
Goat anti-human IgG-HRP antibody	Southern Biotech	Cat#2040-05; RRID: AB_2795644
Goat anti-human IgA (α-chain-specific)-peroxidase antibody	Sigma-Aldrich	Cat#A0295; RRID: AB_257876
Goat anti-human IgM (μ-chain-specific)-peroxidase antibody	Sigma-Aldrich	Cat#A6907; RRID: AB_258318
Goat anti-Rabbit, AlexaFluor 568 conjugated secondary antibody	Thermo Fisher Scientific	Cat#A-11011; RRID: AB_143157
Rabbit anti-SARS-CoV-2 nucleocapsid antibody	Rockland	Cat#200-401-A50; RRID: AB_828403
Mouse anti-human CD4 BV650	BioLegend	Cat#300536; RRID: AB_2632791
Mouse anti-human CD8 APC/Cyanine7	BioLegend	Cat#344714; RRID: AB_2044006
Mouse anti-human CCR7 PE/Cyanine7	BioLegend	Cat#353226; RRID: AB_353226
Mouse anti-human CD3 AF700	BD Biosciences	Cat#561027; RRID: AB_10561682
Mouse anti-human CD45RA	BD Biosciences	Cat# 555489; RRID: AB_395880
Mouse anti-human IFN-γ V450	BD Biosciences	Cat# 560371; RRID: AB_1645594
Mouse anti-human TNF-α PE/Dazzle 594	BioLegend	Cat# 502946; RRID: AB_2564173
Bacterial and virus strains		
SARS-CoV-2/human/Liverpool/REMRQ0001/2020	Dr Lance Turtle	N/A
Biological samples		
Human AB serum	Merck KGaA	Cat#H6914
Chemicals, peptides, and recombinant proteins		
SARS-CoV-2 spike protein	This paper	N/A
SARS-CoV-2 Receptor Binding Domain (RBD) protein	This paper	N/A
SARS-CoV-2 nucleocapsid protein	This paper	N/A
SIGMAFAST OPD (O-phenylenediamine dihydrochloride) tablets	Sigma-Aldrich	Cat#P9187
1-Step Ultra TMB-ELISA Substrate Solution-1 L	Thermo Fisher	Cat#34029
Human recombinant IL-2 protein	R&D Systems	Cat#202-IL
Protein A Sepharose	Cytiva	Cat#17528003
Protein G Sepharose	Cytiva	Cat#17061806
Nano-Glo®	Promega	Cat#N1150
N-terminally nanoluciferase tagged monomeric RBD	Dr Vito Lampasona	N/A
DAPI for nucleic acid staining	Sigma-Aldrich	Cat#D9542-1MG
SARS-CoV-2 spike protein overlapping peptide library (custom made)	Mimotopes	N/A
SARS-CoV-2 spike protein overlapping peptide library (custom made)	Prof Tao Dong	(Peng et al., 2020)[11]
PepTivator SARS-CoV-2 Prot_M-research grade	Miltenyi Biotec	Cat#130-126-702
PepTivator SARS-CoV-2 Prot_N-research grade	Miltenyi Biotec	Cat#130-126-698
PMA	Sigma-Aldrich	Cat#P1585
Ionomycin	Sigma-Aldrich	Cat#I0634
Critical commercial assays		
Expi293 Expression System	Thermo Fisher Scientific	Cat#A14635
Human IFN-γ ELISpot^{PLUS} kit (ALP) strips	Mabtech	3420-4AST-2
Zombie Aqua Fixable Viability Kit	BioLegend	Cat# 423102
OneComp eBeads Compensation Beads	Thermo Fisher Scientific	Cat#01-1111-42

(Continued on next page)

Continued

REAGENT or RESOURCE	SOURCE	IDENTIFIER
Experimental models: cell lines		
Vero E6	ATCC	ATCC® CRL-1586; RRID: CVCL_0574
VeroE6/TMPRSS2	NIBSC	Repository reference: 100978; RRID: CVCL_YQ49
Recombinant DNA		
pFastBacDual spike	Prof Florian Krammer	N/A
pFastBac Dual RBD	Prof Florian Krammer	N/A
pET28a-NP-FL	Prof Ashley Toye	N/A
pCMV-TnT RBD	Dr Vito Lampasona	N/A
Software and algorithms		
Cell ReporterXpress	Molecular Devices	https://www.moleculardevices.com/products/cellular-imaging-systems/acquisition-and-analysis-software/cellreporterxpress#gref
FlowJo V10.7.1	Tree Star, Inc	https://www.flowjo.com; RRID: SCR_008520
GraphPad Prism V9.0	GraphPad	https://www.graphpad.com/scientific-software/prism/; RRID: SCR_002798
R, V 4.0.2	The R Foundation for Statistical Computing	https://www.r-project.org/
R studio, V 1.2.1073	RStudio	https://www.rstudio.com/
Factoextra, V1.0.7	CRAN	https://cran.r-project.org/web/packages/factoextra/index.html; RRID:SCR_016692

RESOURCE AVAILABILITY

Lead contact

Further information and requests for resources and reagents should be directed to and will be fulfilled by the lead contact, Anu Goenka (anu.goenka@bristol.ac.uk).

Materials availability

This study did not generate new unique reagents.

Data and code availability

The datasets generated during this study have been uploaded to https://data.mendeley.com at https://dx.doi.org/10.17632/v78gcvxc2s.3

EXPERIMENTAL MODEL AND SUBJECT DETAILS

Human subjects and samples

Clinical information and blood/saliva samples were obtained under research ethics approval of the Bristol Biobank (NHS REC 14/WA/1253). Written informed consent was obtained from parents and adult control cases. Information regarding donor demographics can be found in Table 1. Pre-pandemic adult serum samples were also obtained from the Bristol Biobank and were used as controls for the serology assays. Blood was collected by venepuncture into EDTA tubes (BD Biosciences) for PBMC isolation by Ficoll gradient [using Leucosep tubes (Greiner Bio-One) when blood volume was sufficient], and SST tubes (BD Sciences) for serum; PBMC and serum samples were stored in liquid nitrogen or at −70°C, respectively, until further use. Serum samples were heat inactivated for 30 minutes at 56°C prior to their use in the assays. Pre-pandemic saliva was collected from adults on a sterile sponge (Malvern Medical Developments) as previously described.[16] Saliva samples were collected from adult study participants into a funnel over a collection tube (Isohelix). Saliva was collected from infants using a sterile oral swab (Iskus Health). Some assays could not be performed on all participants because of the limited blood/saliva volumes available.

Cell lines

Vero E6 cells (ATCC) and Vero E6 cells engineered to express the cell surface protease TMPRSS2 (Vero-TMPRSS2) (National Institute for Biological Standards and Control) were cultured at 37°C in 5% CO_2 in Dulbecco's Modified Eagle's medium containing

GlutaMAX (Thermo Fisher Scientific) supplemented with 10% fetal calf serum (FCS) (Thermo Fisher Scientific) and 0.1 mM non-essential amino acids (NEAA) (Sigma Aldrich).

METHOD DETAILS

Protein production for ELISA

SARS-CoV-2 trimeric spike protein ectodomain and receptor binding domain (RBD) were produced in insect cells as previously described.[17] SARS-CoV-2 spike ectodomain was expressed in insect cells with pFastBac Dual (Thermo Fisher Scientific) plasmid as previously described,[18] a gift from Florian Krammer (Icahn School of Medicine, USA). This construct of spike contains amino acids 1 to 1213 and with a C-terminal thrombin cleavage site, a T4-foldon trimerization domain followed by a hexahistidine tag for affinity purification. In this construct, polybasic cleavage site has been removed (RRAR to A).[18] pFastBac Dual plasmid for insect cell expression of SARS-CoV-2 RBD was also a gift from Florian Krammer. This construct is comprised of spike amino acid 319 to 541, preceded at N terminus with the secretion signal sequence of native spike (MFVFLVLLPLVSSQ) and followed by a c-terminal octa-histidine tag for purification. For both spike and RBD, MultiBac baculovirus expression system was used to produce the proteins in Hi5 insect cells as previously described.[19] A similar purification protocol was used for both spike and RBD. Three days after infection, cell cultures expressing the spike or RBD protein were centrifuged at 1,000 g for 10 min to collect the media with secreted protein as supernatant, which was again centrifuged at 5,000 g for 30 min. This media was then incubated with 7 mL (10 mL for RBD) HisPur Ni-NTA Superflow Agarose (Thermo Fisher Scientific) for each 3 L of expression for 1 hour at 4°C. Next, Ni-NTA resin bound with spike or RBD was collected using a gravity flow column, followed by extensive wash with 15 column volume wash buffer (65 mM NaH2PO4, 300 mM NaCl, 20 mM imidazole, pH 7.5). Finally, a step gradient of elution buffer (65 mM NaH2PO4, 300 mM NaCl, 235 mM imidazole, pH 7.5) was used to elute the protein. Elution fractions were analyzed by reducing SDS-PAGE. Fractions containing spike or RBD were pooled and concentrated using 50 kDa MWCO Amicon centrifugal filter units (EMD Millipore) and then finally buffer-exchanged in phosphate-buffered saline (PBS) pH 7.5 before aliquoting and flash freezing in liquid nitrogen. Samples were stored at −80°C until further use.

A codon-optimized, N-terminal His6 tagged full length nucleocapsid protein of SARS-CoV-2 was synthesized and cloned by GenScript into a pET28a bacterial expression plasmid, (called here pET28a-NP-FL). The pET28a-NP-FL plasmid was transformed into *E. coli* strain BL21 (DE3). Protein expression was induced by the addition of 1 mM IPTG and then incubated overnight at 20°C. Cells were pelleted by centrifugation and resuspended in 20 mM Tris pH 8, 500mM NaCl, 10 mM imidazole, 1 mM NaF and 1 mM PMSF. Cells were lysed by passage through a French Press (Spectronic Instruments) and the resulting lysates were centrifuged at 39,000 g at 4°C for 30 min. The supernatant was applied to a HisTrap HP nickel affinity column (GE Healthcare) and washed using a series of wash buffers containing 10-40 mM Imidazole (20 mM Tris pH 8, 500 mM NaCl, containing 10, 20 and 40 mM Imidazole). The protein was eluted in 20 mM Tris pH 8, 500 mM NaCl and 500 mM imidazole and further purified by size exclusion chromatography using a HiLoad 16/600 Superdex 200 pg column (GE Healthcare) equilibrated and eluted in 20 mM Tris pH 8 and 500 mM NaCl. Peak fractions were pooled and concentrated in a 10 kDa MWCO Vivaspin ultrafiltration unit. Protein concentration was determined using the Bradford assay. Typical yields of N proteins after Ni-NTA and size exclusion chromatography was approximately 9 mg/L. Purified proteins were analyzed by SDS-PAGE and by Western-blot using an anti-his tag antibody (Sigma).

Serum ELISA

Serum antibodies specific for SARS-CoV-2 spike protein, RBD and the nucleocapsid protein were detected by an ELISA based on described methodology.[18] Spike, RBD and nucleocapsid were each diluted in sterile PBS (Sigma) and MaxiSorp plates (NUNC) were coated with either 10 μg/ml (spike) or 20 μg/ml (RBD; nucleocapsid protein) of protein overnight at 4°C before use. Plates were blocked with a 1-hour incubation in 3% Bovine Serum Albumin (BSA) (Sigma-Aldrich) in PBS with 0.1% Tween-20 (Sigma-Aldrich) (PBS-T) at room temperature. Serum samples were thawed before use, tested in duplicate and diluted to a final volume of 100 μl per well at a pre-optimized dilution, either at 1 in 50 (PanIg, IgA, IgM assays) or 1 in 450 dilution (IgG assay), in dilution buffer (1% BSA in PBS-T) and all samples tested on a single plate for each antigen and antibody isotype combination. Secondary antibodies were used as follows with the dilution factor indicated: HRP conjugated anti-human IgG (Southern Biotech: 1 in 25,000), IgA (Sigma: 1 in 6,000-10,000), and IgM (Sigma: 1 in 3,000). SIGMAFAST™ OPD (o-phenylenediamine dihydrochloride) (Sigma-Aldrich) was used to develop plates and reactions were stopped after 30 minutes with 3M HCl. Optical density was read at 492 nm (to measure signal) and 620 nm (background) using a BMG FLUOstar OMEGA PlateReader with MARS Data Analysis software. The optical density (OD) readings at 492 nm for each well were subtracted from the OD at 620 nm then corrected for the average signal of blank wells from the same plate; ODs reported are an average of duplicate wells per sample.

Saliva ELISA

Salivary antibodies specific for SARS-CoV-2 spike protein were detected with an ELISA based on the methodology described above with some modifications. Antigens were diluted in PBS and MICROLON® plates (Griener Bio-One) were coated with 10 μg/mL spike protein overnight at 4°C. Saliva was heat inactivated at 56°C for 30 minutes and centrifuged at 13,000 g for 5 minutes to pellet debris. Saliva supernatants were assayed singly, diluted at either 1 in 10 (IgA) or 1 in 5 (IgG) to a final volume of 100 μL per well. Secondary antibodies were as described for serum with concentrations optimized for saliva: IgA at 1:20,000 and IgG at 1:15,000. Plates were

developed with 1-StepUltra TMB-ELISA Substrate Solution (Thermo Fisher) for 20 minutes and the reaction was quenched with 2M H2SO4 (Merck). All incubations were temperature controlled at 23°C. ODs were read at 450 nm and 570 nm using the same reader.

Luciferase immunoprecipitation system (LIPS)

Detection and quantification of IgG specific to RBD was performed using an N-terminally nanoluciferase tagged monomeric RBD construct with competitive displacement based on previously described methodology.[20] To make the construct, modified coding sequences were designed and obtained as synthetic genes (Eurofins Genomics) allowing production of secretory Nanoluciferase n-terminally tagged RBD domain through subcloning of the antigen into modified pCMV-TnT (Promega) vectors. Recombinant nanoluciferase-tagged antigen was expressed by transient transfection of the corresponding plasmid into Expi293F cells (Expi293 Expression System, Thermo Fisher Scientific) according to the manufacturer's instructions. Recombinant protein was harvested after 48 hours from the supernatant and stored and shipped at −80°C. The procedure for immunoassay was, briefly, samples (1 uL, 4 replicates) were incubated for 2 hours at RT with $4x10^6$ (+/−5%) luminescence units of N-terminally nanoluciferase tagged monomeric RBD construct diluted in 25 uL of buffer (20 mM Tris Buffer, 150mM NaCl, 0.5% Tween-20, pH 7.4 [TBST], and 0.05% casein in label incubation buffer only) with or without addition of unlabelled RBD ($8x10^{-8}$ mol/L). Immunocomplexes were precipitated using 2.5μl glycine-blocked Protein A Sepharose 4 fast flow (Cytiva) and 2.5μl ethanolamine-blocked Protein G Sepharose (Cytiva) (washed 4 times in TBST) for 1hr with shaking (~700rpm) as previously described.[21] Precipitates were washed 5 times with TBST and then transferred to a 96-well OptiplateTM (Perkin-Elmer) and excess buffer removed by aspiration (end volume 30 uL). Nano-Glo® substrate (40 μL, Promega) was injected into each well immediately before counting in a Hidex Sense Beta (Hidex). Raw data were converted into units using a standard curve made by serially diluting a pool of positive samples in SARS-CoV-2 antibody negative human AB serum (Merck KGaA).

Virus neutralisation assay

Heat inactivated serum samples (30 min at 56°C) were serially diluted 2.5-fold, from a 1:20 starting dilution in duplicate in Minimum Essential Media (Thermo Fisher Scientific) containing 2% FBS and NEAA for 8 dilutions. Control wells containing virus only (no sera) as well as positive and negative control sera were also included on each plate. SARS-CoV-2 virus from the isolate SARS-CoV-2/human/Liverpool/REMRQ0001/2020 (gift from Dr. Lance Turtle, University of Liverpool) was grown on Vero-TMPRSS2 cells and titrated as previously described.[22] Virus was mixed with dilutions of the sera at a multiplicity of infection of 0.4 and incubated for 60 min at 37°C. Following the incubation, supernatants were removed from the cells and virus:sera dilutions were added to Vero E6 cells seeded previously in μClear 96 well microplates (Greiner Bio-One) and incubated for 18 hours at 37°C in 5% CO_2. Cells were fixed by incubation in 4% PFA for 60 minutes followed by permeabilisation with Triton X-100 and blocking with BSA. Cells were stained with DAPI (Sigma Aldridge) and an antibody against the SARS-CoV-2 nucleocapsid protein (200-401-A50, Rockland) in combination with a corresponding fluorophore conjugated secondary antibody (Goat anti-Rabbit, AlexaFluor 568, Thermo Fisher Scientific). Images were acquired on the ImageXpress Pico Automated Cell Imaging System (Molecular Devices) using a 10X objective. Stitched images of 9 fields covering the central 50% of the well were analyzed for infected cells using Cell ReporterXpress software (Molecular Devices). Briefly, cell numbers were determined by automated counted of DAPI stained nuclei, infected cells were determined as those cells in which positive nucleocapsid staining, associated with a nucleus, was detected. The percentage of infected cells relative to control wells containing virus only (no sera) were calculated.

Synthetic peptides

15-mer peptides overlapping by ten amino acid residues and spanning the SARS-CoV-2 spike protein were either purchased from Mimotopes (Australia) or donated by Prof Tao Dong (Oxford University). The purity of the peptides were > 80% or > 75%, respectively. Peptides were dissolved as described previously.[23] SARS-CoV-2 membrane and nucleocapsid Peptivator peptide pools were purchased from Miltenyi Biotec.

ELISpot

Cryopreserved PBMCs were thawed and rested in a humidified incubator at 37°C/5% CO_2 for 5 hours. Human IFN-γ ELISpot assays were performed using Human IFN-γ ELISpot^{PLUS} Kit (MABTECH) according to manufacturer's instructions. Pre-coated (mAb-D1K) plates were washed four times in sterile PBS and blocked for 2-3 hours using R10 (RPMI/10% FCS) medium. Rested PBMC were washed, counted, and resuspended in R2 (RPMI/2% FCS) medium; $2x10^5$ PBMC were added to the plate with or without peptide pools (see below) in a total assay volume of 100 μL. PBMC incubated with R2 medium alone were used as negative (unstimulated) controls. PBMC stimulated with PMA at 1 μg/mL and ionomycin at 10 μg/mL (Sigma Aldrich), or anti-CD3 antibody (MABTECH, Mab CD3-2, 0.1% v/v) were used as positive controls (1-$2x10^5$ PBMC per well). Antigen-specific cellular responses were measured following stimulation with an overlapping peptide library spanning the entire spike protein (divided across two pools: S1 and S2) (Mimotopes) at a final concentration of 2 μg/mL in R2. All assays were performed in duplicate. Plates were incubated for 18 hours at 37°C/5% CO_2 in a humidified incubator. For development, plates were washed 5 times in PBS then incubated for 2 hours at room temperature with detection antibody (7-B6-1-biotin; 1μg/mL) in reagent diluent (PBS/0.5% FCS). Following incubation, plates were washed 5 times in PBS and incubated for 1 hour at room temperature with 0.1% v/v Streptavidin-ALP diluted in reagent diluent. Developed plates were protected from light and dried for 24-48 hours before image acquisition using C.T.L. ImmunoSpot S6 Ultra-V

Analyzer. All plates were read using the same settings. Spot forming units (SFU) per million PBMC were calculated after subtraction of average background calculated from negative control wells.

Ex vivo stimulation, intracellular cytokine staining and flow cytometry

Cryopreserved PBMC were thawed, washed and plated at $1x10^6$ cells per well in AIM-V medium (Invitrogen) with 2% FCS in a 96-well plate, and incubated for 5 hours at 37°C/5% CO_2 in the presence of brefeldin A at 5 μg/ml (BD Biosciences) with overlapping peptides from the SARS-CoV-2 proteins spike (see above), membrane (Miltenyi Biotec) and nucleoprotein (Miltenyi Biotec) at 1 μg/ml final concentration, or PMA at 10 ng/mL and ionomycin at 100ng/mL (Sigma Aldrich), or unstimulated (media) control. Following incubation, cells were stained with Zombie Live/Dead Aqua (BioLegend) for 10 min at room temperature before staining for surface markers for 20 min at 4°C, diluted in PBS (HyClone) 1% BSA (Sigma Aldrich) with an antibody cocktail containing anti-CD4 BV650, anti-CD8 APC-Cy7 and anti-CCR7 PE-Cy7 (BioLegend) as well as anti-CD3 AF700 and anti-CD45RA PE (BD Biosciences) antibodies. Cells were then fixed for 45 min in eBioscience Foxp3/Transcription factor fixation/permeabilization buffer (Invitrogen) and intracellular staining was performed for detection of intracellular cytokines [(anti-IFN-γ V450, BD Biosciences), (anti-TNF-α PE/Dazzle 594, BioLegend)] using eBioscience Foxp3/Transcription factor permeabilization buffer for Ki67 (Invitrogen) for 30 min on ice. Cells were acquired on a BD Fortessa X20 cytometer. Single stain controls were prepared using compensation beads (OneComp, Thermo Fisher Scientific). Samples were analyzed after compensation was set using FlowJo (Version 10.3, FlowJo LLC) and gating determined using the fluorescence-minus-one principle. The frequency of cytokine positive cells following stimulation was calculated by subtracting the frequency observed in a well containing cells without exogenous stimuli (i.e., media only) from a parallel well containing stimulated cells.

In vitro expansion of T cells with SARS-CoV-2 peptides

PBMCs were thawed and washed with PBS 1% BSA. 20% of cells were pulsed in AIM-V medium with 2% human serum (Merck KGaA) with peptide pools from SARS-CoV-2 spike protein, membrane/nucleocapsid proteins at 5 μg/ml for 45 min at 37°C 5% CO_2. After stimulation cells were washed in PBS 1% BSA and resuspended with remaining 80% of the PBMCs in AIM-V 2% human serum with 20 IU/ml of IL-2 (R&D Systems) and cultured for 10 days in 96-well U well plates at $0.6x10^6$ cells /well, as previously described.[24] After 10-day expansion culture, cells were re-stimulated for 5 hours with SARS-CoV-2 peptide pools (as described above in *ex vivo* experiments) or unstimulated (media) control. The frequency of cytokine positive cells following re-stimulation was calculated by subtracting the frequency observed in a well containing cells without exogenous re-stimulation (i.e., media only) from a parallel well containing re-stimulated cells.

QUANTIFICATION AND STATISTICAL ANALYSIS

Statistical analysis and plots were produced using Prism (Version 9.0, GraphPad Software). Comparisons of the antibody/cellular response between the infant and parent groups were made using the Mann Whitney U test. When the antibody/cellular responses of infants were compared with more than one adult group, the significance was determined by the Kruskal-Wallis test with Bonferroni's correction for multiple comparisons. The Benjamini-Hochberg method was used to control for the False Discovery Rate (FDR) of multiple assays being performed on the same sample. The significance levels were set at $p < 0.05$ and FDR < 0.05. To investigate variation in immune responses of infants and parents, all features were reduced using principal component analysis (R statistical software version 4.0.2; prcomp function). Data from all antibody and cellular assays were included and scaled. Missing values were imputed with group means for family 1 saliva IgG/IgA and IFNγ production by total PBMCs (ELISpot). Principal components were visualized and 95% confidence ellipses plotted using the factoextra package (Version 1.0.7).

Cell Reports Medicine, Volume *2*

Supplemental information

Young infants exhibit robust functional antibody responses and restrained IFN-γ production to SARS-CoV-2

Anu Goenka, Alice Halliday, Michaela Gregorova, Emily Milodowski, Amy Thomas, Maia Kavanagh Williamson, Holly Baum, Elizabeth Oliver, Anna E. Long, Lea Knezevic, Alistair J.K. Williams, Vito Lampasona, Lorenzo Piemonti, Kapil Gupta, Natalie Di Bartolo, Imre Berger, Ashley M. Toye, Barry Vipond, Peter Muir, Jolanta Bernatoniene, Mick Bailey, Kathleen M. Gillespie, Andrew D. Davidson, Linda Wooldridge, Laura Rivino, and Adam Finn

SUPPLEMENTAL INFORMATION

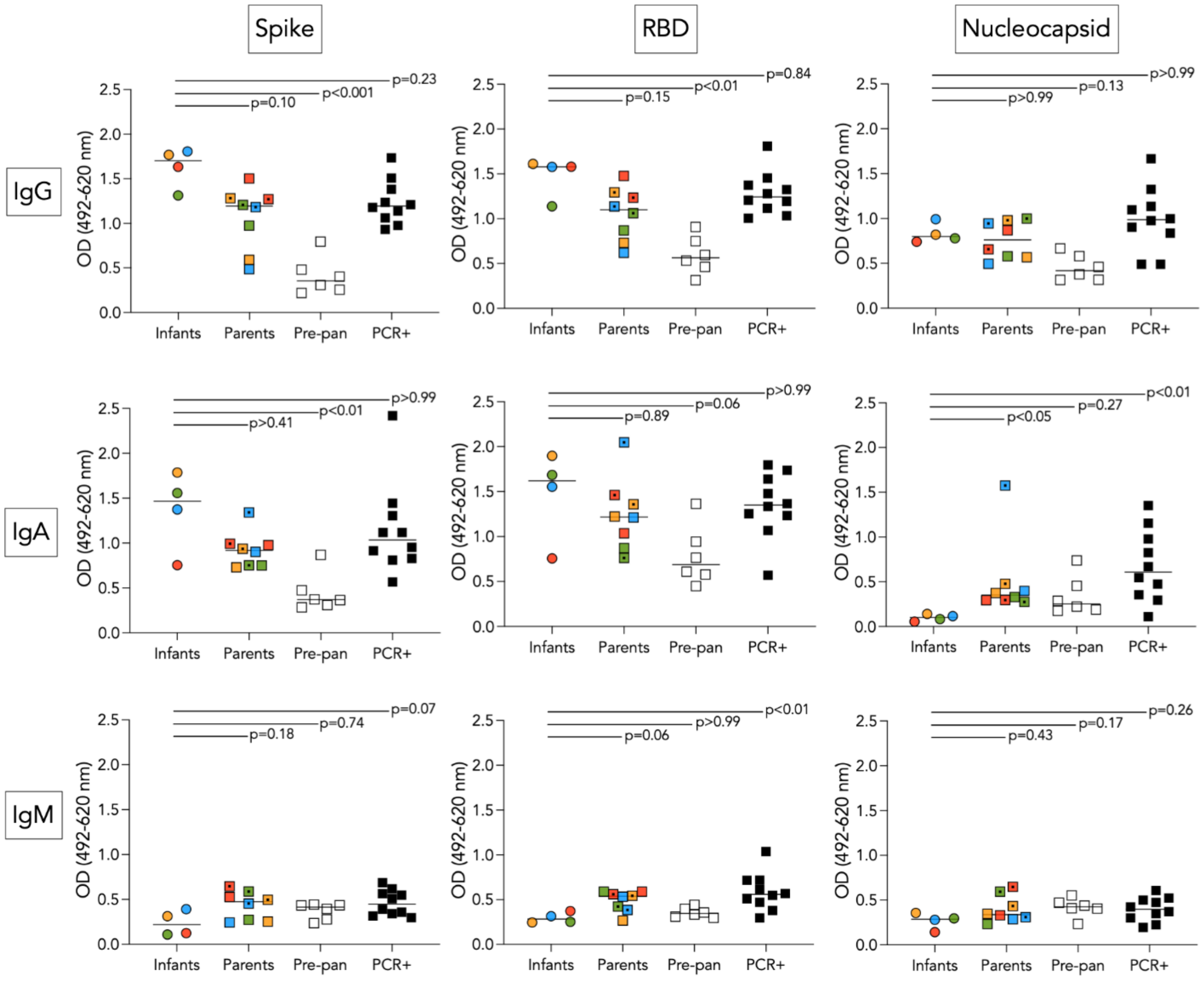

Figure S1. Robust antibody production to SARS-CoV-2 proteins across IgG and IgA isotypes in young infant sera. Related to Figure 1. Measurement of serum IgG, IgA and IgM against SARS-CoV-2 spike protein, receptor-binding domain (RBD) and nucleocapsid protein by ELISA, where background optical density (OD) at 620 nm was subtracted from signal OD at 492 nm.

All data points represent mean of technical duplicates. Individual families denoted by colour (1: red, 2: green, 3: blue; 4: orange); infants (coloured circles), fathers (coloured squares); mothers (coloured square with central marking); RT-PCR confirmed adult COVID-19 controls (black squares); pre-pandemic sera (clear squares). Significance determined by Kruskal-Wallis test with Bonferroni’s correction for multiple comparisons.

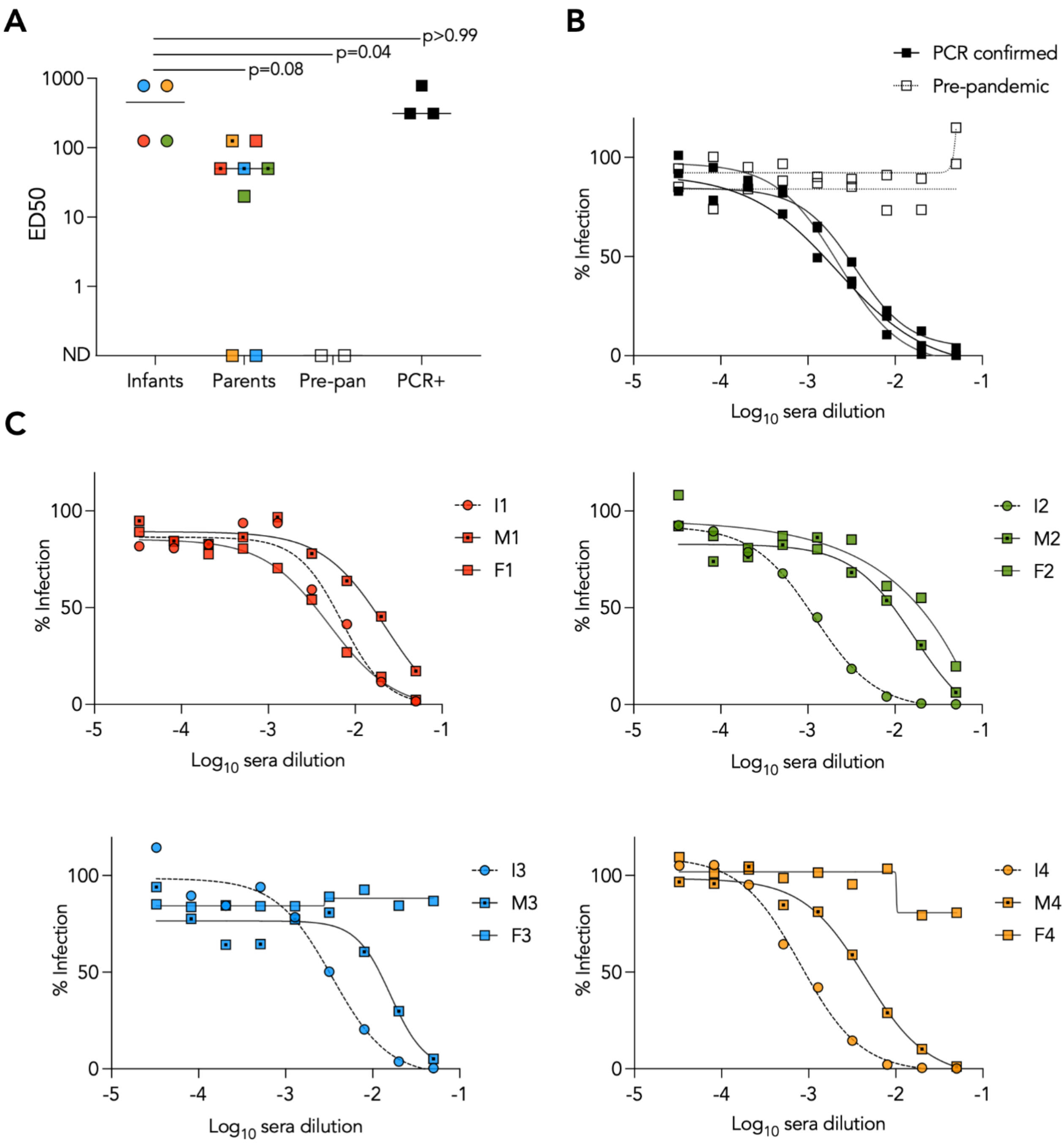

Figure S2. Robust neutralisation of SARS-CoV-2 by young infant sera. Related to Figure 1. (**A**) Serum neutralisation expressed as half maximal effective dose/dilution (ED50) of sera to achieve SARS-CoV-2 neutralisation. Significance determined by Kruskal-Wallis test with Bonferroni's correction for multiple comparisons; (**B**) Neutralisation of SARS-CoV-2 by serial dilutions of sera from pre-pandemic and RT-PCR confirmed adult controls; and (**C**) sera from each individual family. Neutralisation of SARS-CoV-2 measured by infection of Vero E6 cells with SARS-CoV-2 pre-incubated with decreasing concentrations of serum, where % infection is shown relative to virus-only control.

All data points represent mean of technical duplicates. Individual families denoted by colour (1: red, 2: green, 3: blue; 4: orange); infants (coloured circles), fathers (coloured squares); mothers (coloured square with central marking); RT-PCR confirmed adult controls (black squares); pre-pandemic controls (clear squares).

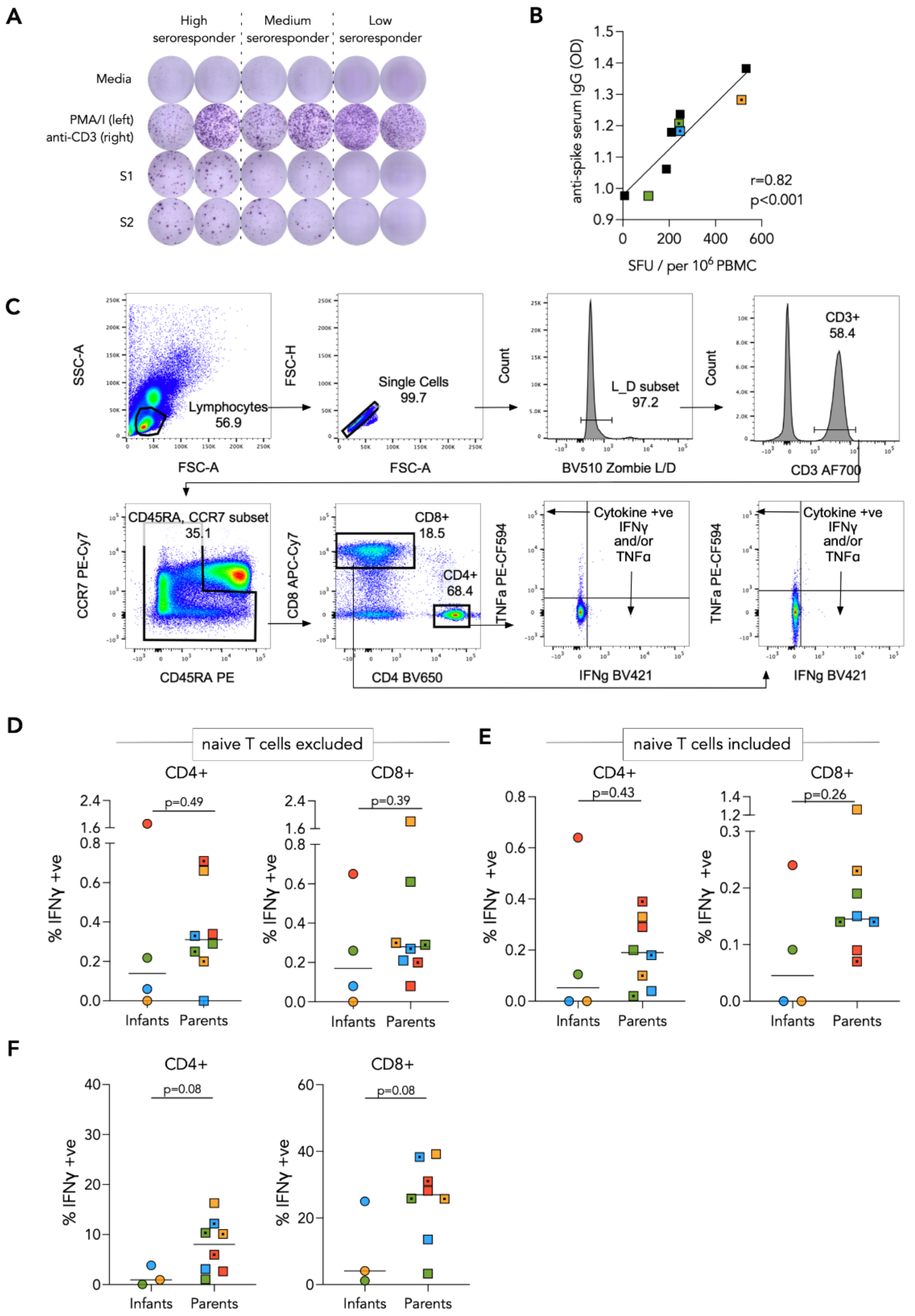

Figure S3. Detection of *ex vivo* cytokine production from PBMCs stimulated with SARS-CoV-2 peptide pools. Related to Figure 2. (**A**) Representative image of an ELISpot plate following 18h stimulation with SARS-CoV-2 peptide pools (S1, S2 at 2µg/mL), unstimulated (media) or positive control stimulus (PMA 1µg/mL/Ionomycin 10µg/mL or anti-CD3 antibody 0.1% v/v) of PBMCs from adults recovered from RT-PCR confirmed COVID-19; (**B**) Correlation of IFN-γ production measured by ELISpot and anti-spike IgG measured by ELISA in seropositive adults; (**C**) flow cytometry gating strategy for intracellular cytokine staining (ICS); (**D**) and (**E**) IFN-γ production of CD4+ and CD8+ T cells (with and without naïve CD45RA$^+$ CCR7$^+$ included respectively) measured by ICS of PBMCs following 5h *ex vivo* stimulation with SARS-CoV-2 peptide pools spanning spike (S1/S2) (1µg/mL). Significance determined by Mann Whitney U test**; (F)** IFN-γ production of CD4+ and CD8+ T-cells measured by ICS of PBMCs after 5h stimulation with PMA 1µg/mL/Ionomycin 10µg/mL. Significance determined by Mann Whitney U test.

Data points represent single observations (ICS) or mean of technical duplicates (ELISpot) with unstimulated signal subtracted in both ELISpot and ICS assays. Individual families denoted by colour (1: red, 2: green, 3: blue; 4: orange); infants (coloured circles), fathers (coloured squares); mothers (coloured square with central marking); and RT-PCR confirmed adult COVID-19 controls (black squares).
